# Targeting Bile Acid Receptors: Discovery of a Potent and Selective Farnesoid X Receptor Agonist as a New Lead in the Pharmacological Approach to Liver Diseases

**DOI:** 10.3389/fphar.2017.00162

**Published:** 2017-03-30

**Authors:** Carmen Festa, Simona De Marino, Adriana Carino, Valentina Sepe, Silvia Marchianò, Sabrina Cipriani, Francesco S. Di Leva, Vittorio Limongelli, Maria C. Monti, Angela Capolupo, Eleonora Distrutti, Stefano Fiorucci, Angela Zampella

**Affiliations:** ^1^Department of Pharmacy, University of Naples “Federico II”Naples, Italy; ^2^Department of Surgery and Biomedical Sciences, Nuova Facoltà di MedicinaPerugia, Italy; ^3^Institute of Computational Science – Center for Computational Medicine in Cardiology, Faculty of Informatics, Università della Svizzera ItalianaLugano, Switzerland; ^4^Department of Pharmacy, University of SalernoFisciano, Italy; ^5^Ospedale S. Maria della Misericordia, Azienda Ospedaliera di PerugiaPerugia, Italy

**Keywords:** bile acids, bile acid receptors, farnesoid X receptor, drug discovery, liver-disorders, cholestasis, fibrosis

## Abstract

Bile acid (BA) receptors represent well-defined targets for the development of novel therapeutic approaches to metabolic and inflammatory diseases. In the present study, we report the generation of novel C-3 modified 6-ethylcholane derivatives. The pharmacological characterization and molecular docking studies for the structure-activity rationalization, allowed the identification of 3β-azido-6α-ethyl-7α-hydroxy-5β-cholan-24-oic acid (compound **2**), a potent and selective FXR agonist with a nanomolar potency in transactivation assay and high efficacy in the recruitment of SRC-1 co-activator peptide in Alfa Screen assay. *In vitro*, compound **2** was completely inactive towards common off-targets such as the nuclear receptors PPARα, PPARγ, LXRα, and LXRβ and the membrane G-coupled BA receptor, GPBAR1. This compound when administered *in vivo* exerts a robust FXR agonistic activity increasing the liver expression of FXR-target genes including *SHP, BSEP, OSTα*, and *FGF21*, while represses the expression of CYP7A1 gene that is negatively regulated by FXR. Collectively these effects result in a significant reshaping of BA pool in mouse. In summary, compound **2** represents a promising candidate for drug development in liver and metabolic disorders.

## Introduction

Bile acids (BAs), the end products of cholesterol metabolism, are increasingly recognized for their role as signaling molecules. The signaling pathways involve the interaction with several nuclear receptors (NRs) and cell surface G-protein-coupled receptors (G-PCRs), including the G protein-coupled bile acid receptor GPBAR1 (also known as TGR5 or M-BAR) (Maruyama et al., 2002; Kawamata et al., 2003; Fiorucci and Distrutti, 2015; Copple and Li, 2016). Among NRs, the farnesoid X receptor (FXR) (Makishima et al., 1999; Parks et al., 1999; Wang et al., 1999) is the master gene that orchestrates BA homeostasis. Upon BA binding, FXR regulates a network of genes in synthesis, uptake, and secretion along with intestinal absorption, thus regulating the level of BAs in the cells (Goodwin et al., 2000). An abnormal BA metabolism associates with liver injury, metabolic disorders, cardiovascular and digestive system diseases (Fiorucci and Baldelli, 2009; Fiorucci et al., 2010b, 2014; Sepe et al., 2015a).

In addition, FXR plays a crucial beneficial role in triglyceride and cholesterol homeostasis, as well as in glucose metabolism (Fiorucci et al., 2004, 2007, 2009, 2010a,c; Cariou et al., 2006; Zhang et al., 2006; Cipriani et al., 2010; Mencarelli et al., 2013; Swanson et al., 2013).

As a consequence, FXR ligands have been claimed as new therapeutical options in a wide range of diseases related to metabolic, inflammatory and immune-modulated disorders including type II diabetes, primary biliary cirrhosis (PBC), nonalcoholic fatty liver disease (NAFLD), and nonalcoholic steatohepatitis (NASH) (Fiorucci and Baldelli, 2009; Fiorucci et al., 2011b, 2012a,b, 2014; Sepe et al., 2015a).

On the other hand, GPBAR1 activation exerts useful pharmacological effects such as increased energy expenditure by brown and white adipose tissue, glucagon-like peptide 1 (GLP-1) secretion by intestinal endocrine cells which hold the potential for beneficial effects on glucose metabolism and insulin sensitivity (vanNierop et al., 2017; Hodge and Nunez, 2016).

Collectively, these findings have prompted the development of dual GPBAR1/FXR agonists as a new frontier in the pharmacological treatment of hypercholesterolemia, hypertriglyceridemia, and type II diabetes (Fiorucci et al., 2009; Fiorucci and Distrutti, 2015; Sepe et al., 2015b). However, the concomitant activation of GPBAR1 associates with potential side effects, including itching (Alemi et al., 2013; Lieu et al., 2014), cholesterol gallstone formation (Vassileva et al., 2006) and gallbladder overfilling (Li et al., 2011). Therefore, the discovery of highly selective FXR agonists, devoid of GPBAR1 agonist activity, is therapeutically attractive for the treatment of conditions where the concomitant activation of GPBAR1 might increase the risk for adverse side effects.

**FIGURE 1:**
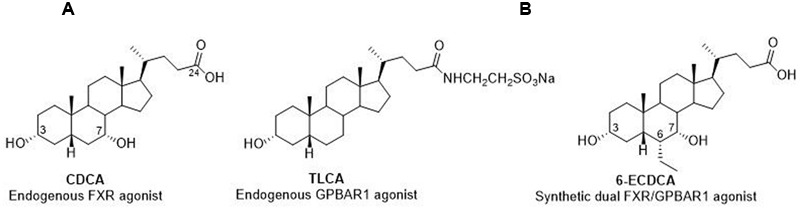
**Endogenous and semisynthetic bile acids as FXR and GPBAR1 agonists. (A)** CDCA and TLCA, the endogenous activators of FXR and GPBAR1, respectively. **(B)** 6-ECDCA, a semisynthetic dual agonist.

The activity of BAs towards their receptor counterparts is structure dependent, with chenodeoxycholic acid (CDCA) and tauro-lithocholic acid (TLCA), being the most potent endogenous activators of FXR and GPBAR1 (**Figure 1A**), respectively.

In recent years, we have reported the chemical manipulation on CDCA scaffold, with the aim to improve potency, efficacy and metabolic stability of endogenous BAs, affording several hit compounds with promising pharmacological profiles (Di Leva et al., 2015; Sepe et al., 2016; Finamore et al., 2016).

In detail, the introduction of an ethyl group at C-6 on the CDCA afforded to the disclosure of 6-ECDCA/OCA (**Figure 1B**)/INT-747 endowed with high potency toward FXR (Pellicciari et al., 2002). 6-ECDCA has been widely investigated in *in vitro* and *in vivo* (Fiorucci et al., 2011a) and in a phase III clinical trial in patients with nonalcoholic steatohepatitis (NASH) (Neuschwander-Tetri et al., 2015). Despite 6-ECDCA improved several features of NASH, including inflammation and fibrosis, the above positive findings were tempered by the appearance of pruritus in 23% of patients and by an increase in total cholesterol and LDL. In addition, administration in PBC patients caused pruritus in approx. 50–60% that was severe enough to cause drug discontinuation in 40% of patients (Mason et al., 2010). Indeed 6-ECDCA is also a ligand for GPBAR1 (Festa et al., 2014; Pellicciari et al., 2016; Sepe et al., 2016) and therefore the above side effect might be associated to the activation of the membrane BA receptor, recently demonstrated bona fide to be the physiological mediator of itching in mice (Alemi et al., 2013; Lieu et al., 2014).

**FIGURE 2:**
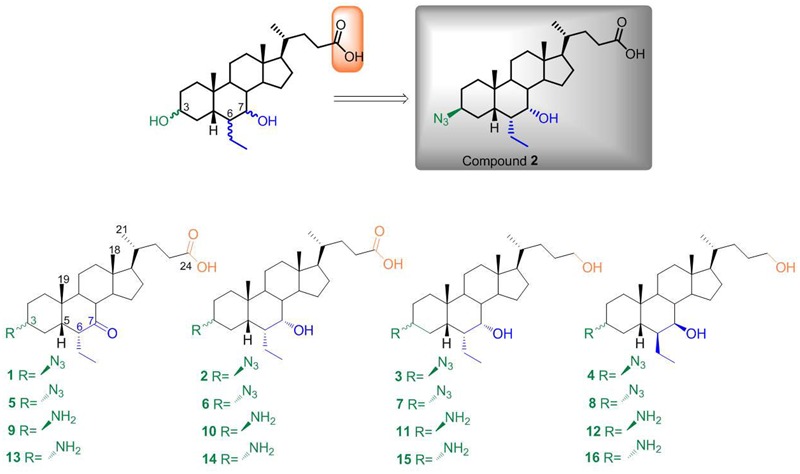
**Chemical library prepared in this study.** Modification at C-3, C-6, C-7, and C-24 on 6-ethylcholane scaffold and identification of compound **2** as the best hit in this series.

In the present work, we have modified 6-ECDCA scaffold installing an azido/amino group at the C-3 position. The *rationale* for this modification is based on our recent demonstration that the 3α-OH on BAs forms a stable H-bond with a negatively charged residue (Glu169) in GPBAR1 (D’Amore et al., 2014; Di Leva et al., 2015) whereas in FXR-LBD the above functional group interacts with a positively charged residue (His444). Therefore, the introduction at C-3 of a polarizable group (dipole) bearing a partial negative charge on the ligand atom interacting with the receptor residues, could represent a good strategy to shift the activity towards FXR. In order to explore further the chemical space, we manipulated also the side chain and the configurational assessment of the ethyl group at C-6 and the hydroxyl group at C-7, producing the small library reported in the **Figure 2**. Among this library, optimized compound **2** represents a FXR agonist with a nanomolar potency (EC_50_ = 846 nM) in transactivation assay and high efficacy in the recruitment of SRC-1 co-activator peptide in Alfa Screen assay. The above potency was accompanied by high selectivity with compound **2** devoid of any activity toward common off-targets such as the NRs LXRα/β and PPARα/γ and the cell surface G-PCR GPBAR1. Further, *in vivo* pharmacological characterization demonstrated that compound **2** represses BA synthesis in the liver through the regulation of FXR targeted gene expression. Collectively, these data, combined with the good pharmacokinetic behavior, affirm compound **2** as a new therapeutical opportunity for the treatment of liver FXR-mediated diseases.

## Materials and Methods

### Chemical Material

All reactions were carried out under argon atmosphere using flame-dried glassware. Solvents and reagents were used as supplied from commercial sources with the following exceptions. Hexane, ethyl acetate, chloroform, dichloromethane, tetrahydrofuran and triethylamine were distilled from calcium hydride immediately prior to use. Methanol was dried from magnesium methoxide. Reaction progress was monitored via thin-layer chromatography (TLC) on Alugram silica gel G/UV254 plates. Silica gel MN Kieselgel 60 (70–230 mesh) from Macherey–Nagel Company was used for column chromatography. All chemicals were obtained from Sigma-Aldrich, Inc. The purity of tested compounds was determined to be always greater than 95% by analytical HPLC analysis (Waters Model 510 pump equipped with Waters Rheodine injector and a differential refractometer, model 401) using a Nucleodur 100-5 C18 Isis (5 μm; 4.6 mm i.d. ×250 mm). High-resolution ESI-MS spectra were performed with a Micromass Q-TOF mass spectrometer. NMR spectra were obtained on Varian Inova 400, 500, and 700 NMR spectrometers (^1^H at 400, 500, and 700 MHz,^13^C at 100, 125, and 175 MHz, respectively) equipped with a SUN microsystem ultra5 hardware and recorded in CD_3_OD (δ_H_ = 3.31 and δ_C_ = 49.0 ppm) and CDCl_3_ (δ_H_ = 7.26 and δ_C_ = 77.0 ppm). All of the detected signals were in accordance with the proposed structures. Coupling constants (*J* values) are given in Hertz (Hz), and chemical shifts (δ) are reported in ppm and referred to CHD_2_OD and CHCl_3_ as internal standards. Spin multiplicities are given as s (singlet), br s (broad singlet), d (doublet), t (triplet), or m (multiplet). For details on synthetic procedures, see the Supplementary Material.

### Alpha Screen Assay

Activation of FXR was determined by Alpha Screen Technology in a Coactivator Recruitment Assay. Anti-GST-coated acceptor beads were used to capture the GST-fusion FXR-LBD, whereas the biotinylated-SRC-1 peptide was captured by the streptavidin donor beads. Upon illumination at 680 nm, chemical energy is transferred from donor to acceptor beads across the complex streptavidin-donor/SRC-1-biotin/GSTFXR-LBD/anti-GST-acceptor and a signal is produced. The assay was performed in white, low-volume, 384-well Optiplates (PerkinElmer) using a final volume of 25 μL containing final concentrations of 10 nM of purified GST-tagged FXR-LBD protein, 30 nM biotinylated SRC-1 peptide, 20 μg/mL anti-GST acceptor beads, and 10 μg/mL of streptavidin donor bead (PerkinElmer). The assay buffer contained 50 mM Tris (pH 7.4), 50 mM KCl and 1 mM DTT. The stimulation times with 1 μL of tested compound (dissolved in 50% DMSO/H_2_O) were fixed to 30 min at room temperature. The concentration of DMSO in each well was maintained at a final concentration of 2%. After the addition of the detection mix (acceptor and donor beads), the plates were incubated in the dark for 3 h at room temperature and then were read in an Envision microplate analyzer (PerkinElmer).

### Transactivations on HepG2 Cells

HepG2 (HB, 8065 from ATCC), an immortalized human epatocarcinoma cell line, was cultured and maintained at 37°C and 5% CO_2_ in E-MEM additioned with 10% FBS, 1% glutamine, and 1% penicillin/streptomycin. For FXR mediated transactivation, HepG2 cells were plated at 5 × 10^4^ cells/well in a 24 well plate. Cells were transfected with 200 ng of the reporter vector p(hsp27)-TK-LUC containing a FXR response element (IR1) cloned from the promoter of heat shock protein 27 (hsp27), 100 ng of pSG5-FXR, 100 ng of pSG5-RXR, and 100 ng of pGL4.70 (Promega), a vector encoding the human Renilla gene. For GPBAR1 mediated transactivation, HepG2 cells were plated at 5 × 10^4^ cells/well in a 24 well-plate and transfected with 200 ng of pGL4.29 (Promega), a reporter vector containing a cAMP response element (CRE) that drives the transcription of the luciferase reporter gene luc2P, with 100 ng of pCMVSPORT6-human GPBAR1, and with 100 ng of pGL4.70 Renilla. In both assays, at 24 h post-transfection, cells were stimulated 18 h with compounds **1–16** (1 and 10 μM). 6-ECDCA (1 and 10 μM) was used as a positive control for FXR activity. TLCA (10 μM) was used as a positive control for GPBAR1 activity.

### *In vitro* Selectivity of Compound **2**

To evaluate LXRα and LXRβ mediated transactivation, HepG2 cells were transfected with 200 ng of the reporter vector p(UAS)5XTKLuc, 100 ng of a vector containing the ligand binding domain of LXRα or LXRβ cloned upstream of the GAL4-DNA binding domain (i.e., pSG5-LXRαLBD-GAL4DBD or pSG5-LXRβLBD-GAL4DBD) and 100 of pGL4.70 Renilla. At 24 h post-transfection, cells were stimulated 18 h with 10 μM compound **2** and GW3965 (GW, 10 μM) was used as positive control. To investigate the PPARα and PPARγ mediated transactivation, HepG2 cells were transiently transfected with 200 ng reporter vector p(UAS)9XTKLuc, 100 ng of a vector containing the ligand binding domain of PPARα or PPARγ cloned upstream of the GAL4-DNA binding domain (i.e., pSG5- PPARαLBD-GAL4DBD or pSG5- PPARγLBD-GAL4DBD) and 100 of pGL4.70 Renilla. For PPARα transactivation, cells were stimulated with 10 μM compound **2** and gemfibrozil (GEM, 10 μM) was used as positive control. For PPARγ transactivation, cells were stimulated with 10 μM compound **2** and rosiglitazone (ROSI, 100 nM) was used as a positive control. Luciferase activities were assayed and normalized with Renilla activities.

### Dose-Response Curve for Compound **2** and Its Tauro-Conjugate 2a on FXR

HepG2 cells were transfected as described above and then treated with increasing concentrations of compounds. At 18 h post stimulations, cellular lysates were assayed for luciferase and Renilla activities using the Dual-Luciferase Reporter assay system (E1980, Promega). Luminescence was measured using Glomax 20/20 luminometer (Promega). Luciferase activities were normalized with Renilla activities.

### RNA Isolation and RT-PCR

HepG2 cells were plated at 1 × 10^6^ cells/well in a six well plate. After an overnight incubation, cells were starved and then stimulated for 18 h with compound **2** at 1 μM. Total RNA was isolated from HepG2 cells or liver tissues using the TRIzol reagent according to the manufacturer’s specifications (Invitrogen). One microgram of purified RNA was treated with DNase-I and reverse transcribed with Superscript II (Invitrogen). For Real Time PCR, 10 ng template was dissolved in 25 μL containing 200 nmol/L of each primer and 12.5 μL of 2× SYBR FAST Universal ready mix (Invitrogen). All reactions were performed in triplicate, and the thermal cycling conditions were as follows: 2 min at 95°C, followed by 40 cycles of 95°C for 20 s and 60°C for 30 s in StepOnePlus (Applied Biosystems). The relative mRNA expression was calculated accordingly to the Ct method. Primers were designed using the software PRIMER3^1^ using published data obtained from the NCBI database. Forward and reverse primer sequences were the following: human *GAPDH*, gaaggtgaaggtcggagt and catgggtggaatcatattggaa; human *BSEP*, gggccattacgagatccta and tgcaccgtcttttcactttctg; human *OSTα*, tgttgggccctttccaatac and ggctcccatgttctgctcac; human *SHP*, tctcttcttccgccctatca and aagggcttgctggacagtta; mouse *NTCP*, ggtgccctacaaaggcatta and gttgcccacattgatgacag; mouse *CYP7A1*, aagccatgatgcaaaacctc and gccggaaatacttggtcaaa; mouse *FGF21*, acacagatgacgaccaagacac and aagtgaggcgatccatagagag; mouse *GAPDH*, ctgagtatgtcgtggagtctac and gttggtggtgcaggatgcattg; mouse *BSEP*, atgcttgtgaccctgcaaa and agatcgttgacggatggaag; mouse *OSTα*, ctttggtgggaagaaagcag and gaagaaggcgtactggaaagg; mouse *SHP*, tctcttcttccgccctatca and aagggcttgctggacagtta.

### Animal

C57BL6 male mice were from Harlan Nossan (Udine, Italy). The colonies were maintained in the animal facility of University of Perugia. Mice were housed under controlled temperatures (22 °C) and photoperiods (12:12-hour light/dark cycle), allowed unrestricted access to standard mouse chow and tap water and allowed to acclimate to these conditions for at least 5 days before inclusion in an experiment. A total number of eight mice were used in this study. The study was conducted in agreement with the Italian law and the protocol was approved by Ethical Committee of University of Perugia and by a National Committee of Ministry of Health (permission N° 42/2014 B). The health and body conditions of the animals were monitored daily by the veterinarian in the animal facility. At the day of sacrifice mice were deeply anesthetized with a mixture of tiletamine hypochlorite and zolazepam hypochlorite/xylazine at a dose of 50 mg/Kg.

### Animal Models

C57BL6 male mice (8) were administered with compound **2** (50 mg/Kg body weight per os) or vehicle (distilled water) for 3 days. At the day of sacrifice livers, gallbladders, blood and feces were collected from mice for further analysis.

### Bile Acid Determinations

Bile acids pools were measured by liquid chromatography-tandem mass spectrometry (MS/MS) analysis, using chromatographic conditions as described elsewhere (John et al., 2014). The stock solutions of the individual tauro-conjugated and un-conjugated BAs were prepared separately in methanol at a concentration of 1 mg/mL. All stock solutions were stored at –20°C. Calibration standards were prepared by combining appropriate volumes of each BA stock solution and methanol. The calibration range was from 10 nM to 100 μM of each BA in the final solution. Mice serum sample aliquots of 20 μL were mixed with 80 μL of CH_3_OH, shaken continuously, vortexed and, after centrifugation at 16000 *g* for 10 min, the clear supernatant was transferred to a new vial, snap frozen and lyophilized. The sample was then re-dissolved in 40% water/ 60% MeOH with 0.1% formic acid and ammonium acetate 5 mM. A BA extraction yield of 95% has been measured using BA standard addition in plasma sample before and after deproteinization procedure. For gallbladder, 10 mg of lyophilized gallbladder were manually pestle using a mortar and dissolved in 1 mL CH_3_OH. After centrifugation at 16000 *g* for 10 min, 500 μL of supernatants were lyophilized and reconstituted in 100 μL of 40% water/60% MeOH with 0.1% formic acid and ammonium acetate 5 mM.

### Liquid Chromatography and Mass Spectrometry

For LC-MS/MS analysis, chromatographic separation was carried out on the HPLC-MS system Q-TRAP 6500 LC-MS/MS System from AB Sciex equipped with Shimadzu LC-20A LC and AutoSampler system. The mixture was separated on a Synergi Fusion RP 4 m from Phenomenex (150 mm × 2.00 mm).

Tauro-conjugated and non-conjugated BAs were separated at a flow rate of 200 μL/min using a methanol–aqueous ammonium acetate (NH_4_OAc) gradient. Mobile phase A was water containing 5 mM ammonium acetate and 0.1% formic acid, mobile phase B was methanol, containing ammonium acetate at 5 mM and 0.1% formic acid. The gradient started at 65% B and increased to 85% B in 23 min, kept at 85% B for 5 min then decreased to 65% B in 1 min and kept at 65% B for 10 min. ESI was performed in negative ion mode and the ion source temperature was set at 280°C. The tune page parameters were automatically optimized injecting taurocholic acid at 1 μM as standard. The MS/MS detection was operated in MRM mode using a collision energy of 20 (arbitrary units) and the observed transitions are reported in Mencarelli et al., 2013.

### Molecular Modeling

In order to investigate ligand interaction with FXR-LBD, we performed docking calculations that are very widely used to generate and rank binding complexes based on empirical scoring functions (Anzini et al., 2008, 2011; Heckmann et al., 2009; Cerqueira et al., 2015). In the present case, we used the Glide (version 7.1) (Schrödinger, 2016a) software package to perform molecular docking calculations in the crystal structure of the FXR-LBD from *Rattus norvegicus* (rFXR) in complex with 6-ECDCA and the GRIP-1 coactivator peptide NID-3 (PDB code 1osv) (Mi et al., 2003). rFXR-LBD shares indeed the 95% of homology with that of the human FXR-LBD (hFXR-LBD), with all of the residues in the ligand binding pocket conserved among the two species. Protein structure was prepared as described in a previous paper (Di Leva et al., 2013). Ligand tridimensional structures were generated with the Maestro build panel (Schrödinger, 2016c). For each ligand, an extensive ring conformational sampling was performed with MacroModel (version 11.3) (Schrödinger, 2016d) using the OPLS3 force field (Harder et al., 2016) and a 2.0 Å rmsd cutoff for clustering. All conformers were then refined using LigPrep (Schrödinger, 2016b) as implemented in Maestro. Protonation states at pH 7.4 were assigned using Epik (Shelley et al., 2007; Greenwood et al., 2010). In Glide, a box of 25 Å × 25 Å × 25 Å centered on the FXR binding cavity was initially created to compute the interaction grids. Upon docking calculations, ligands macrocyclic rings were treated as rigid; otherwise, default parameters were applied. The standard precision (SP) mode of the GlideScore function was used to score and rank the predicted binding poses (Friesner et al., 2004; Halgren et al., 2004). For each ligand, the best 10 docking poses were considered for visual inspection. All the residue labels were taken from the aforementioned crystal structure of rFXR-LBD.

### Statistical Analysis

Statistical analysis was performed with Prism 6.0 software (GraphPad). The non parametric Mann–Whitney *U* test or a 2-tailed unpaired Student *t*-test was used for statistical comparisons (^∗^*P* < 0.05, ^∗∗^*P* < 0.005, ^∗∗∗^*P* < 0.0005).

## Results

### Chemistry

**FIGURE 3:**
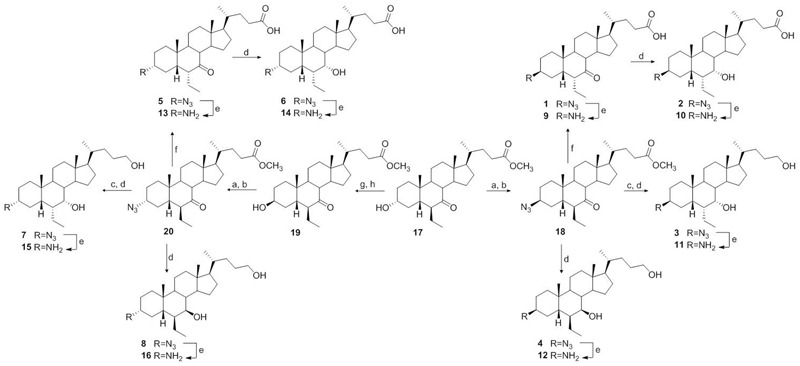
**Preparation of chemical library.** *Reagent and conditions*: (a) MsCl, TEA; ethyl ether, –10°C; (b) NaN_3_, DMSO, DMF, 150°C; (c) NaOMe, MeOH, r.t.; (d) LiBH_4_, MeOH dry, THF, 0°C; (e) NH_4_Cl, Zn, MeOH:H_2_O (1:0.1); (f) NaOH 5% in MeOH/H_2_O 1:1 v/v, reflux; (g) *p*TsCl, pyridine, r.t.; (h) CH_3_COOK, DMF:H_2_O (5:1), reflux.

Our planned strategy started from methyl 6β-ethyl-7-ketocholanoate **17** (**Figure 3**), which was prepared following our previously described procedure (Festa et al., 2014). Mesylation at C-3 and subsequent treatment with NaN_3_ furnished the 3β-azido derivative **18** as a cornerstone intermediate in the preparation of derivatives **1–4**. First, inversion at C-6 with NaOMe/MeOH treatment followed by concomitant reduction at C-7 carbonyl group and at C-24 methyl ester furnished **3**. Basic treatment (NaOH, MeOH/H_2_O) on **18** proceeded in a straightforward manner affording the concomitant hydrolysis on the side chain methyl ester and inversion at C-6 ethyl group producing **1**, that in a small amount was reduced affording the corresponding 7α-hydroxy derivative, compound **2**, in 98% yield after purification. Finally, hydride reduction of **18** afforded pure **4**.

The counterpart 3α-azido derivatives **5–8** were prepared following the synthetic protocol depicted in **Figure 3**. Tosylation at C-3 hydroxyl group on methyl ester **17** followed by inversion of configuration afforded the 3β-hydroxy derivative **19**, which was in turn transformed in the corresponding 3α-azido derivative **20** in the same operative conditions reported for **18** (**Figure 3**). Elaboration of the functional groups on ring B and on the side chain following the same synthetic protocol of the corresponding 3β-azido derivatives afforded compounds **5–8** in good chemical yield. Finally, by reduction of the azido group of the resulting derivatives **1–8** with zinc powder (Lin et al., 2002), the required 3β- and 3α-amino derivatives **9–16** were available (**Figure 3**).

### Cell-Free Alpha Screen Assay on the Whole Library

Potency and efficacy of compounds **1–16** were firstly evaluated on FXR in a cell-free Alpha Screen assay in comparison with 6-ECDCA (**Table 1**).

**Table 1:** FXR activities of compounds **1**–**16** measured as recruitment of SRC-1 co-activator peptide in Alfa Screen assay^a^.

Compound	EC_50_	Efficacy
6-ECDCA^b^	0.12 ± 0.01	100
1		32
2	0.61 ± 0.09	90
3	0.55 ± 0.08	55
4		39
5		24
6	1.38 ± 0.35	61
7	1.80 ± 0.21	57
8		49
9	n.d.	n.d.
10	n.d.	n.d.
11	n.d.	n.d.
12	n.d.	n.d.
14	n.d.	n.d.
15	n.d.	n.d.
16	n.d.	n.d.

*^a^Data represent mean values ± SD of three different experiments.*

*Units are μM for EC_50_. Efficacy is calculated as % of the effect in the recruitment respect to 6-ECDCA at 2 μM.*

*EC_50_ has been reported for efficacy ≥50%; n.d. = non-detectable up to 20 μM*

*^b^6-ECDCA was prepared as previously reported (Finamore et al., 2016).*

In this assay, compound **2** shows a potent activity and high efficacy in the recruitment of SRC-1 peptide, as evidenced by its nanomolar potency at FXR (EC_50_ 610 nM, 90% efficacy respect to 6-ECDCA). Of interest, the above activity decreases with the modification of the configuration at C-3 (compare **2** with the corresponding 3α-azido **6** in **Table 1**) as well as with the introduction of an alcoholic function as side chain end group (compare **2** vs. **3** and **6** vs. **7** in **Table 1**). As expected (Festa et al., 2014), in this subset the configurations at C-6/C-7 as well as the presence of a hydroxyl group at C-7 profoundly affect FXR activation with compounds **1, 4, 5**, and **8** showing a remarkable reduction in term of efficacy in the recruitment of SRC-1 co-activator peptide in Alfa Screen assay. Finally, the corresponding 3-amino derivatives **9–16** did not show any effect in the recruitment assay in the concentration range 20 nM–20 mM.

### Transactivation Assay on HepG2 Cells Transiently Transfected with hFXR

**FIGURE 4:**
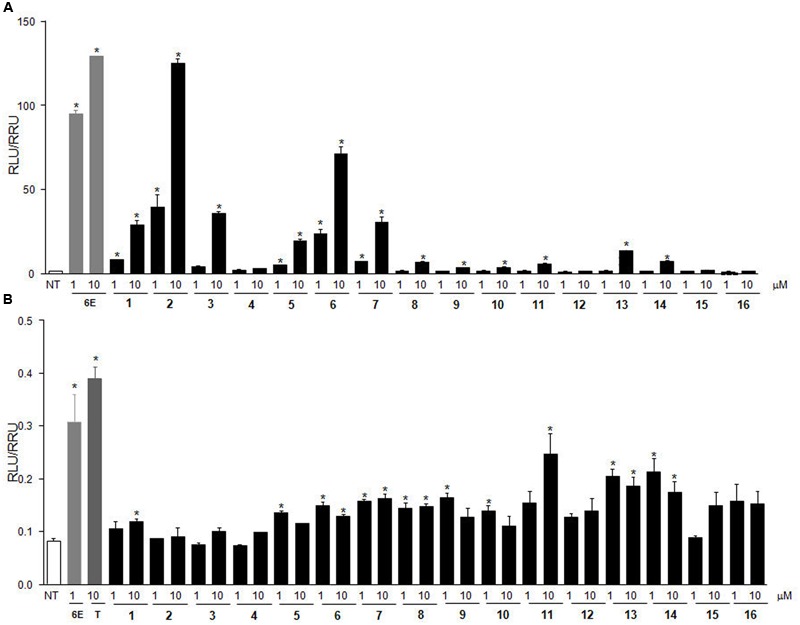
***In vitro* screening on the library. (A)** FXR transactivation on HepG2 cells. HepG2 cells were transfected with pSG5-FXR, pSG5-RXR, PGL4.70-Renilla, and p(hsp27) TKLUC vectors. Cells were stimulated with compounds **1**–**16** (1 and 10 μM). 6-ECDCA (6E, 1 and 10 μM) was used as positive control. **(B)** GPBAR1 transactivation on HepG2 cells. HepG2 cells were co-transfected with GPBAR1 and a reporter gene containing a cAMP responsive element in front of the luciferase gene. Cells were stimulated with 6-ECDCA (6E, 1 μM) and compounds **1–16** (1 and 10 μM). TLCA (10 μM) was used as positive control. Luciferase activity served as a measure of the rise in intracellular cAMP following activation of GPBAR1. In both panels, results are expressed as mean ± standard error. ^∗^*p* < 0.05 *versus* not treated cells (NT).

The above results were substantially confirmed in a transactivation assay on HepG2 cells transiently transfected with hFXR (**Figure 4A**). As reference compound, 6-ECDCA was used in concentrations of 1 and 10 μM. The best results were obtained with compound **2**, which turned out to be equipotent with 6-ECDCA at 10 μM, followed, in order of efficacy at 10 μM, by its 3α-epimer, compound **6**, and the corresponding C-24 alcohol, compound **3**.

### Transactivation Assay on HepG2 Cells Transiently Transfected with GPBAR1

Of interest, results of transactivation of CREB-responsive elements in HepG2 cells, transiently transfected with the membrane BA receptor GPBAR1 (**Figure 4B**), clearly showed that 6-ECDCA is endowed with GPBAR1 agonism at 1 μM whereas the introduction of an azido group at C-3 (compounds **1–8**) is detrimental for GPBAR1 activation. Among the corresponding 3-amino derivatives (compounds **9–16**), compounds **11, 13**, and **14**, even if less potent than TLCA, the endogenous GPBAR1 agonist, showed a residual activity toward the membrane BA receptor. This result is in agreement with our computation model of GPBAR1 (D’Amore et al., 2014) and with our recent observation that the replacement of the 3α-OH on LCA scaffold with a positively charged group should lead to a selective GPBAR1 activation over FXR (Di Leva et al., 2015).

### Computational Studies

**FIGURE 5:**
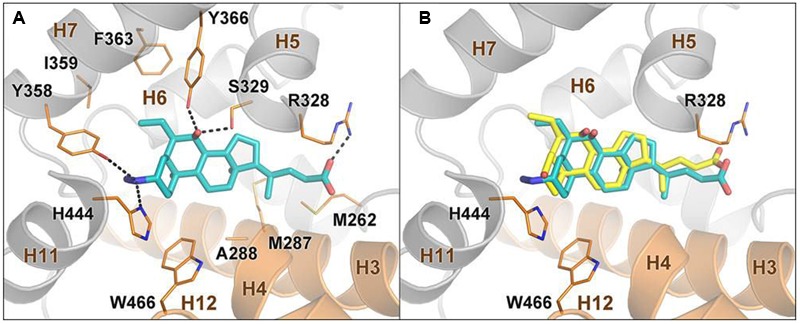
**Docking analysis on compound **2** and 6-ECDCA in FXR ligand binding domain. (A)** Binding mode of compound **2** in the FXR-LBD. The ligand is depicted as cyan sticks, while FXR is shown as orange (helices H3, H4, and H12) and gray cartoons. Amino acids important for ligand binding are shown as orange sticks. Hydrogens are omitted for clarity. **(B)** Superposition between the predicted binding mode pose of **2** and the crystallographic pose of 6-ECDCA (yellow sticks).

Docking calculations, performed using the software GLIDE (Schrödinger, 2016a), allowed the rationalization of the above activities. In particular, the most active compound of the series, compound **2**, was docked in the crystal structure of the FXR ligand binding domain (FXR-LDB) (PDB code 1osv) (Mi et al., 2003) where the binding site is shaped by five alpha-helices H3, H5, H7, H11, and H12. In the best docking pose, the steroidal scaffold of **2** establishes favorable hydrophobic interactions with the side chains of Leu284, Met287, Ala288, Met325, and Leu345, while the ligand carboxylate group forms a salt bridge with Arg328 (**Figure 5A**). The presence of the latter interaction might play a key role in the activation of the receptor as previously suggested in literature (Mi et al., 2003). On the other side, the 3β-azido moiety of **2** engages H-bond interactions with Tyr358 on H7 and His444 on H11. The latter H-bond allows the stabilization of the cation-π interaction formed by His444 and Trp466 on H12, which is crucial to lock FXR in the conformation competent for the recruitment of coactivator peptides and the activation of the transcription of target genes (Mi et al., 2003; Di Leva et al., 2013). Finally, the ligand 7α-OH group forms H-bonds with the Ser329 and the Tyr366 hydroxyl groups, while the 6α-ethyl substituent engages hydrophobic contacts with Tyr358, Ile359, and Phe363, which altogether further stabilize the ligand binding mode. It is also interesting to note that the docking pose of **2** is highly superimposable (**Figure 5B**) with that experimentally found for 6-ECDCA (Mi et al., 2003). Although the configuration of the azido compound is beta at C-3, hence inverted if compared to 6-ECDCA, the geometry of the azido group allows, however, the proper orientation of the ligand in the LBD, pointing towards His444. Furthermore, the dipole moment of the azido group charges negatively the distal nitrogen atom at C-3, thus stabilizing the interaction with the positively charged His444.

Docking simulations on compounds **3, 6** and **7** (see Figure S1 in the Supporting Information) revealed that alcohol derivatives **3** and **7** binds FXR similarly to the carboxylic analogs **2** and **6**, albeit establishing weaker interactions with Arg328. Thus, a polar uncharged group on the BA side chain is tolerated, however the related compounds might show a decreased activity. On the other hand, the oxidation of the 7α-OH group (**1**) and the inversion of configuration at C-6 and C-7 (**4**) weaken the H-bond interactions with Ser329 and Tyr366 and might generate steric clash with the protein residues, leading to less potent analogs. Similarly, the inversion of configuration at C-3 changes the geometry of the H-bond interactions formed by the azido group with the Tyr358 and His444 side chains, weakening the ligand/receptor interaction and thus explaining loss in efficacy shown by the 3α derivatives **5**–**8** compared to the 3β analogs **1**–**4**. Finally, in line with our previous findings (Di Leva et al., 2015), the presence of a protonable primary amine at C-3, such as in derivatives **9**–**16**, is not tolerated due to repulsive electrostatic interactions with the positively charged His444, thus explaining why these compounds are inactive towards FXR.

### Further Pharmacological Characterization of Compound **2**, the Best Hit Generated in this Study

#### Compound **2** Transactivates FXR in a Dose-Dependent Manner

**FIGURE 6:**
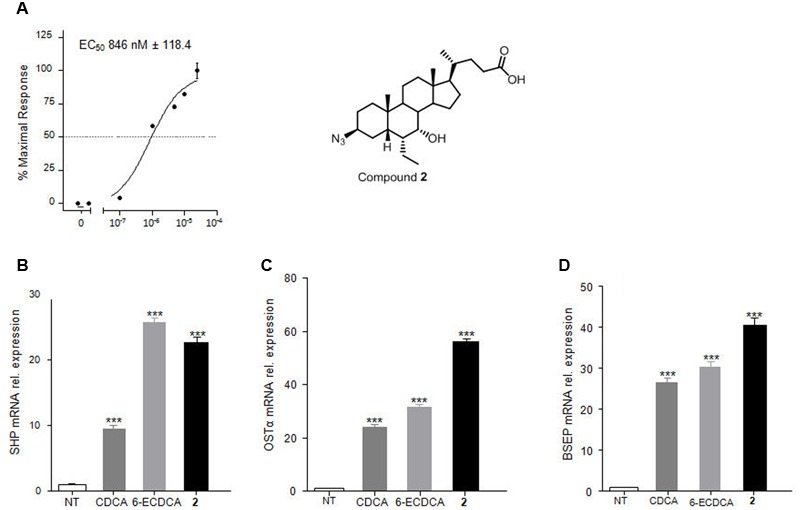
**Compound **2**, the best hit generated in this study.** *In vitro* evaluation on FXR activity. **(A)** FXR activity was measured in HepG2 cells co-transfected with pSG5-FXR, pSG5-RXR, PGL4.70-Renilla, and p(hsp27)TKLUC vectors. Twenty-four hours post transfection cells were stimulated with increasing concentrations of **2** from 10 nM to 25 μM. Results are expressed as mean ± standard error. **(B–D)** RT-PCR analysis of mRNA expression on FXR target genes *SHP* **(B)**, *OSTα* **(C)** and *BSEP* **(D)** in HepG2 cells primed with **2** (1 μM). CDCA (10 μM) and 6-ECDCA (1 μM) were used as positive controls. Values are normalized to *GAPDH* and are expressed relative to those of not treated cells (NT) which are arbitrarily settled to 1. The relative mRNA expression is expressed as 2^(-ΔΔCt)^. ^∗^*p* < 0.05, ^∗∗^*p* < 0.005, ^∗∗∗^*p* < 0.0005 *versus* not treated cells (NT).

The relative potency of compound **2** was first investigated by a detailed measurement of concentration-response curve in transactivation assay on HepG2 cells. As illustrated in **Figure 6A**, compound **2** transactivates FXR with an EC_50_ of 846 nM.

#### Compound **2** Modulates FXR Target Gene Expression in HepG2 Cells

Further, the effect of **2** in modulating FXR target genes was assessed in liver carcinoma cell line HepG2 by RT-PCR, with 6-ECDCA (1 μM) and CDCA (10 μM) as reference compounds. As showed in **Figures 6B–D**, compound **2** at 1 μM concentration resulted more potent than 6-ECDCA in modulating *OSTα* and *BSEP* expression and equipotent with 6-ECDCA in the modulation of *SHP* mRNA expression. Because these three genes are endowed with canonical FXR-responsive elements in their promoter, their induction is fully consistent with the nature of compound **2** as potent FXR agonist.

#### Compound **2** is a Selective FXR Agonist

**FIGURE 7:**
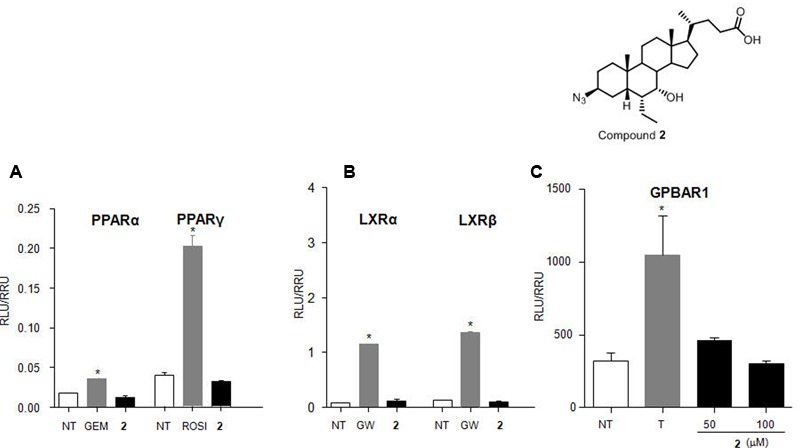
**Target selectivity of compound **2**. (A)** HepG2 cells were transiently transfected with p(UAS)9XTKLuc, pSG5-PPARα-LBD-GAL4DBD or pSG5- PPARγ LBD-GAL4DBD and pGL4.70 Renilla vectors. Cells were stimulated with gemfibrozil (GEM, 10 μM) and rosiglitazone (ROSI, 100 nM), as positive controls for PPARα and PPARγ, respectively, and compound **2** (10 μM). **(B)** HepG2 cells were transfected with p(UAS)5XTKLuc, pSG5-LXRα LBD-GAL4DBD or pSG5-LXRβ LBD-GAL4DBD and pGL4.70 Renilla vectors. Cells were stimulated with GW3965 (GW, 10 μM) as positive control and compound **2** (10 μM). **(C)** HepG2 cells were co-transfected with GPBAR1 and a reporter gene containing a cAMP responsive element in front of the luciferase gene. Cells were stimulated with **2** (50 and 100 μM). TLCA (10 μM) was used as positive control. Luciferase activity served as a measure of the rise in intracellular cAMP following activation of GPBAR1. In all panels, results are expressed as mean ± standard error. ^∗^*p* < 0.05 *versus* not treated cells (NT).

To characterize the pharmacological profile of **2**, we also investigated the activity on common off-targets. As showed in **Figure 7**, compound **2** (10 μM) was unable in inducing PPARα and PPARγ as well as LXRα and LXRβ transactivation on HepG2 cells. Further, **Figure 7C** confirmed the inactivity of **2** toward GPBAR1 at 50 and 100 μM concentrations.

#### Compound **2** Affects Gene Expression in C57BL6 Mice

**FIGURE 8:**
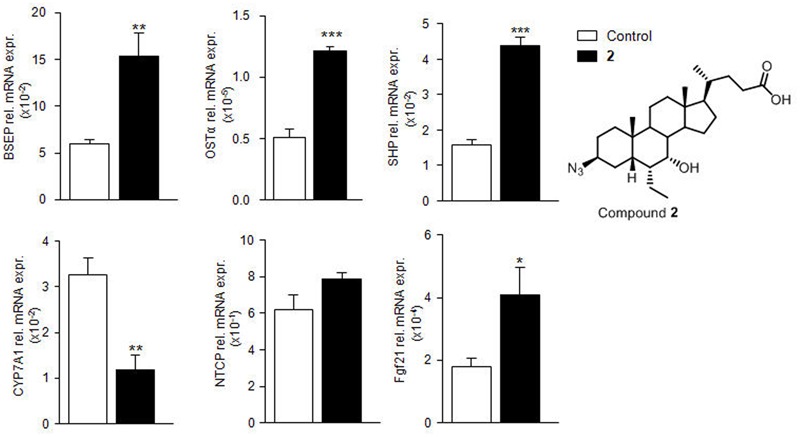
***In vivo* evaluation of compound **2** effect on FXR target gene expression.** C57BL6 mice were treated for 3 days with compound **2** (50 mg/kg per *os*). The relative hepatic mRNA expression of FXR target genes *BSEP, OSTα, SHP, CYP7A1, NTCP, and FGF21* was assayed by RT-PCR. Results are the mean ± SE of 3–5 mice per group. ^∗^*p* < 0.05, ^∗∗^*p* < 0.005, ^∗∗∗^*p* < 0.0005 *versus* naive mice (Control).

Further investigating its pharmacological properties, compound **2** was administered (50 mg/kg, *os*) to C57BL6 mice. At 6 days post-treatment, livers were collected. As shown in **Figure 8**, compound **2** significantly up-regulated the relative mRNA expression of canonical FXR molecular targets such as *BSEP, OSTα, SHP*, and *FGF21* and downregulated *CYP7A1* in the liver (^∗^*p* < 0.05 *versus* control mice), thus confirmed RT-PCR data on HepG2 cells (**Figures 6B–D**).

#### Compound **2** Affects BA Pool in C57BL6 Mice

**FIGURE 9:**
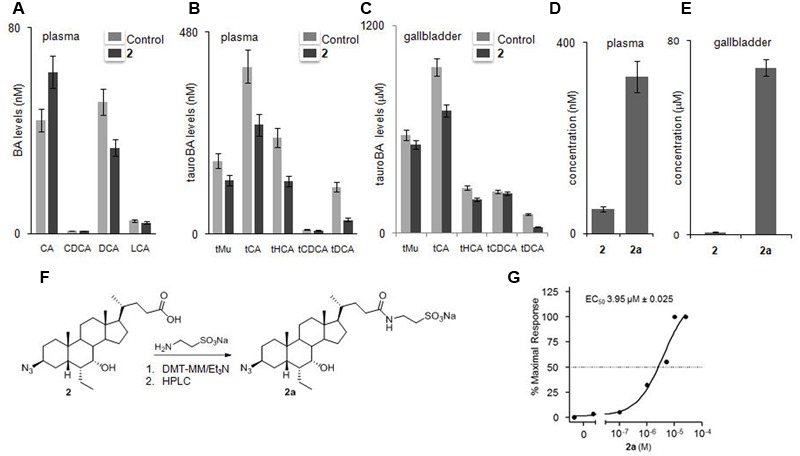
**Effects of compound **2** after mice administration on bile acid pool and evaluation of metabolic profile. (A–C)** Effects of compound **2** administration on levels of unconjugated and tauro-conjugated bile acids in plasma (**A,B**, respectively) and gallbladder **(C)**. **(D,E)** Plasmatic and gallbladder levels of **2** and **2a**. Results are the mean ± SE of 5 mice per group. ^∗^*p* < 0.05 *versus* naive mice, #*p* < 0.05 *versus* **2** treated mice. **(F)** Preparation of compound **2a**. **(G)** *In vitro* evaluation of **2a** on FXR. Activity was measured in HepG2 cells co-transfected with pSG5-FXR, pSG5-RXR, pGL4.70 Renilla and p(hsp27) TKLUC vectors. Twenty-four hours post transfection cells were stimulated with increasing concentrations of **2a** from 10 nM to 25 μM. Results are expressed as mean ± standard error.

Analysis of unconjugated and tauro-conjugated BA concentrations and evaluation of metabolite profile of **2** after oral administration of compound **2** at 50 mg/kg in C57BL6 mice was performed by LC-MS. As showed in **Figures 9B,C**, *in vivo* administration of **2** significantly reduced plasmatic and gallbladder levels of tauro-cholic acid (tCA) and tauro-muricholic acid (tMu), two primary BAs in mouse. The reduction of tCA concentration, together with the reduction of the corresponding secondary BA, tauro-deoxycholic acid (tDCA), is consistent with FXR agonistic profile of compound **2** and the consequent downregulation of hepatic enzymes involved in BA synthesis, such as cytochrome P450 7A1 (CYP7A1).

### *In vivo* Metabolic Profile of Compound **2**

Evaluation of the pharmacokinetic and metabolite profiles of compound **2** first required the preparation of the corresponding tauro-conjugate derivative **2a** through amidation with taurine followed by HPLC purification (**Figure 9F**). LC-MS analysis demonstrated a large grade of tauro-conjugation in the liver with compound **2a** efficiently recovered in bile and in plasma (**Figures 9D,E**). Transactivation assay demonstrated the preserved dose dependent agonistic activity on FXR with an EC_50_ value in micromolar range and comparable to that of free carboxylic acid **2** (**Figure 9G**). Because endogenous BAs and semisynthetic BA derivatives are extensively conjugated in the liver (tauro-conjugation in mice and glyco-conjugation in human), this result highlights the therapeutical potential of **2** in human FXR mediated diseases.

## Discussion

FXR is a BA sensor. In hepatocytes, a rise in intracellular BA concentrations results in the transcriptional activation of FXR. One FXR target gene is the small heterodimer partner (*SHP*), whose transcriptional activation results in a decrease in *CYP7A1*, the key enzyme in BA synthesis, gene expression and therefore in the inhibition of BA synthesis through the neutral pathway (Wang et al., 2002).

In this paper, we report the generation of BA derivatives characterized by the installation of an azido/amino group at the C-3 position of 6-ethylcholane scaffold. A member of this family of compounds, and namely compound **2**, appears to be specific for FXR and does not activate other NRs including LXRα and β and PPARα and γ. Compound **2** is endowed with potent activity toward FXR in cell free assay (EC_50_ 610 nM) and in transactivation assay on HepG2 cells (EC_50_ 846 nM), while the compound is essentially devoid of any activity toward GPBAR1.

The *in vitro* characterization of compound **2** in HepG2 cells demonstrated that this agent exerts FXR agonistic activity and increases the expression of three canonical FXR targeted genes, such as *SHP, OSTα* and *BSEP*. As pointed before, SHP is an orphan NR that lacks a DNA binding domain and plays an essential role in FXR signaling in target cells. SHP functions as a co-repressor for *CYP7A1* leading to a robust inhibition of the synthesis of endogenous BAs in the liver. Although a SHP-independent mechanism exists that negatively regulates BA synthesis, in the context of FXR signaling, induction of SHP represents a robust measure of FXR activation. Indeed, all three genes shown in this study to be regulated by compound **2**, are directly modulated by FXR through its binding to canonical FXR-responsive elements in their promoter.

The fact that compound **2** behaves as FXR ligand was confirmed *in vivo*. Indeed, administration of compound **2** to mice resulted in a profound reshaping in the expression of FXR target genes in the liver. The results shown in **Figure 8** demonstrate that compound **2** when fed to mice at the dose of 50 mg/kg increases the expression of *BSEP, OSTα* and *SHP* mRNAs while represses the gene expression of *CYP7A1*. Taken together these data are consistent with concept that compound **2** activates FXR and represses the synthesis of endogenous BAs. In addition, compound **2** increases the gene expression of *FGF21*. Because, FGF21 is thought to act in an autocrine fashion by binding to the FGF receptor 4 and regulating BA synthesis via repression of *CYP7A1* gene expression, these data strongly indicate that compound **2** is a robust inhibitor of the synthesis of endogenous BAs (Kliewer and Mangelsdorf, 2015). This view is strongly supported by results shown in **Figures 9A–C**. Indeed, administering mice with compound **2** effectively reduced the level of tauro-conjugated BAs in the blood and gallbladder. The blood levels of three primary BAs, i.e., tCA, tMu, and tHCA, were significantly reduced by treating mice with compound **2** at the dose of 50 mg/kg. These findings were completely consistent with the repression of *CYP7A1* gene expression in the liver. These data were also confirmed by examining the relative concentrations of the above tauro-conjugated primary BAs in the gallbladder.

The preliminary characterization of *in vivo* pharmacokinetic properties of compound **2** revealed that this compound undergoes an extensive liver metabolism (**Figures 9D,E**). Thus, while compound **2** is partially excreted as intact molecule in the bile and could be found in the gallbladder and blood, we observed that this compound is mostly disposed by the liver as a tauro-conjugate. Importantly, the tauro-derivative of compound **2**, i.e., compound **2a**, maintains a full agonist activity toward FXR (**Figure 9G**).

FXR ligands have been exploited in recent years in the treatment of a variety of human diseases (Fiorucci and Baldelli, 2009; Fiorucci et al., 2011b, 2012a,b, 2014; Fiorucci, 2012; Sepe et al., 2015a). The most extensive characterization has been in the treatment of PBC and steatohepatitis. Despite the prototype of this class, 6-ethylCDCA (6-ECDCA) also known as obeticholic acid, has shown some effectiveness in the treatment of these conditions, the severity of itching represents a significant limitation to its use. A proportion of approximately 50–60% of PBC patients (Mason et al., 2010) and 23% of patients with NASH (Neuschwander-Tetri et al., 2015) develop itching when treated with obeticholic acid. While the reason for this effect has not been elucidated, there is evidence that it could be linked to the activation of GPBAR1 (Festa et al., 2014; Pellicciari et al., 2016; Sepe et al., 2016), which is considered an itching receptor (Alemi et al., 2013; Lieu et al., 2014). Thus, development of highly selective FXR ligands might help to overcome this limitation.

In summary, we have discovered a novel family of selective FXR ligands that regulate the expression of FXR target genes in the liver and repress BA synthesis. These compounds might hold utility in the treatment of FXR-mediated diseases.

## Author Contributions

CF, SDM, VS, and AZ designed and performed synthesis; ADC, SM, SC, ED, and SF designed and performed *in vitro* and *in vivo* pharmacological experiments; FDL and VL designed and performed computational studies; MM and ANC performed bile acid determination and Alfa Screen assay. All authors contributed to manuscript writing, read and approved the final version.

## Conflict of Interest Statement

The authors declare that the research was conducted in the absence of any commercial or financial relationships that could be construed as a potential conflict of interest.

## Footnotes

1 http://frodo.wi.mit.edu/primer3/
