# Supplementary material for: Targeting Bile Acid Receptors: Discovery of a Potent and Selective Farnesoid X Receptor Agonist as a New Lead in the Pharmacological Approach to Liver Diseases
Source: Front Pharmacol. 2017 Mar 30;8:162. doi: 10.3389/fphar.2017.00162 (PMC5371667; doi:10.3389/fphar.2017.00162)
Supplement: Supplementary file 1 [file Presentation_1.PDF]

Supplementary Material

Targeting bile acid receptors: discovery of a potent and selective Farnesoid X receptor agonist as a new lead in the pharmacological approach to liver diseases

Carmen Festa, Simona De Marino, Adriana Carino, Valentina Sepe, Silvia Marchianò, Sabrina Cipriani, Francesco Saverio Di Leva, Vittorio Limongelli, Maria Chiara Monti, Angela Capolupo, Eleonora Distrutti, Stefano Fiorucci, and Angela Zampella*

***Correspondence:** Angela Zampella: angela.zampella@unina.it

1 Supplementary Data
1.1 Synthetic procedures S2

2 Supplementary Figures and Tables
2.1 Supplementary Figure 1. S12

1.1 Synthetic procedures

Compound 17. Compound methyl 3α-hydroxy-6β-ethyl-7-keto-5β-cholan-24-oate (**17**) was prepared as previously reported (Festa et al., 2014).

Methyl 3β-azido-6β-ethyl-7-keto-5β-cholan-24-oate (18). To a solution of **17** (400 mg, 0.93 mmol) in dry ethyl ether (5 mL), mesyl chloride (360 μL, 4.6 mmol) and TEA (640 μL, 4.6 mmol) were added, and the mixture was stirred at -10 °C for 1 h and then was poured into saturated $NaHCO_3$ solution (10 mL) and extracted with ethyl ether (3 × 10 mL). The combined organic layers were washed with water (10 mL), and then dried over anhydrous $MgSO_4$ and evaporated *in vacuo* to give 480 mg of methyl 3α-mesyloxy-6β-ethyl-7-keto-5β-cholan-24-oate (quantitative yield), that was subjected to next step without any purification. The intermediate (480 mg, 0.94 mmol) was dissolved in dry DMSO (5 ml) and sodium azide (610 mg, 9.4 mmol) was added. The mixture was stirred vigorously at 150 °C for 12 h, and then partitioned between water and ethyl acetate (10 ml). The organic phases were dried (Na_2SO_4), filtered and concentrated *in vacuo* to give 380 mg of methyl 3β-azido-6β-ethyl-7-keto-5β-cholan-24-oate (**18**) (89%). Selected ^{1}H NMR (400 MHz, $CDCl_3$): δ_H 3.77 (1H, br s, H-3), 3.61 (3H, s, -$COOCH_3$), 1.19 (3H, s, H_3-19), 0.87 (3H, d, J = 6.3 Hz, H_3-21), 0.62 (3H, t, J = 7.1 Hz, H_3-26), 0.62 (3H, s, H_3-18); ^{13}C NMR (100 MHz, $CDCl_3$): δ_C 215.7, 174.6, 60.9, 57.6, 54.8, 51.4, 48.7, 45.5, 45.1, 43.1, 42.5, 40.9, 38.7, 35.7, 35.1, 33.2, 30.9 (2C), 28.1, 26.4, 26.1, 24.8, 24.0, 21.6, 18.3, 12.9, 12.0. HR ESIMS *m/z* 458.3387 $[M + H]^+$, $C_{27}H_{44}O_3N_3$ requires 458.3383.

3β-azido-6α-ethyl-7-keto-5β-cholan-24-oic acid (1). Compound **18** (200 mg, 0.44 mmol) was hydrolysed with NaOH (180 mg, 4.4 mmol) in a solution of MeOH:H_2O 1:1 v/v (5 mL) overnight under reflux. The resulting solution was then concentrated under vacuum, diluted with water, acidified with HCl 6 N and extracted with ethyl acetate (3 x 10 mL). The collected organic phases were washed with brine, dried over Na_2SO_4 and evaporated under reduced pressure to give 180 mg of compound **1** as white solid (92%). An analytic sample was obtained by HPLC on a Nucleodur

100-5 C18 Isis (5 μm; 4.6 mm i.d. x 250 mm) with MeOH/H_2O (90:10) as eluent (flow rate 1 mL/min, t_R= 18 min). Selected ^{1}H NMR (500 MHz, CD_3OD): δ_H 3.90 (1H, br s, H-3), 2.86 (1H, m, H-6), 2.50 (1H, t, *J* = 11.3 Hz, H-8), 2.33 (1H, m, H-23a), 2.21 (1H, m, H-23b), 1.27 (3H, s, H_3-19), 0.96 (3H, d, *J* = 6.5 Hz, H_3-21), 0.81 (3H, t, *J* = 7.5 Hz, H_3-26), 0.70 (3H, s, H_3-18); ^{13}C NMR (100 MHz, CD_3OD): δ_C 215.6, 178.0, 59.1, 56.4, 52.8, 51.3, 50.5, 47.8, 45.0, 43.9, 40.3, 37.2, 36.5, 32.3, 32.0, 31.1, 29.2, 26.8, 25.6, 25.0, 24.4, 23.2, 19.7, 18.9, 12.5, 12.0. HR ESIMS *m/z* 442.3072 [M - H], $C_{26}H_{40}O_3N_3$ requires 442.3070.

3β-amino-6α-ethyl-7-keto-5β-cholan-24-oic acid (9). To a solution of **1** (30 mg, 67.7 x 10^{-3}mmol) in methanol (2.5 mL) and water (250 μL) were added solid ammonium chloride (36 mg, 0.68 mmol) and zinc powder in portions (66 mg, 1.01 mmol). After stirring overnight, the reaction mixture was filtered through a short pad of Celite and concentrated. The resulting residue was diluted with ethyl acetate, washed with brine, dried over Na_2SO_4 and evaporated under reduced pressure. HPLC purification on a Nucleodur 100-5 C18 Isis (5 μm; 4.6 mm i.d. x 250 mm) with MeOH/H_2O (60:40) as eluent (flow rate 1 mL/min), gave 15 mg of 3β-amino-6α-ethyl-7-keto-5β-cholan-24-oic acid (**9**) as white solid (53%, t_R= 25.5 min). Selected ^{1}H NMR (400 MHz, CD_3OD): δ_H 3.48 (1H, br s, H-3), 2.91 (1H, m, H-6), 2.54 (1H, t, *J* = 11.6 Hz, H-8), 2.22 (1H, m, H-23a), 1.33 (3H, s, H_3-19), 0.96 (3H, d, *J* = 7.0 Hz, H_3-21), 0.83 (3H, t, *J* = 7.6 Hz, H_3-26), 0.71 (3H, s, H_3-18); ^{13}C NMR (100 MHz, CD_3OD): δ_C 215.1, 178.2, 56.5, 52.6, 51.2, 50.4, 49.8, 46.6, 44.6, 43.8, 40.3, 37.3, 37.0, 35.9, 33.8, 29.8, 29.3, 26.2, 25.6, 24.2, 24.0, 23.1, 19.9, 19.1, 12.5, 12.2. HR ESIMS *m/z* 416.3170 [M - H], $C_{26}H_{42}O_3N$ requires 416.3165.

3β-azido-6α-ethyl-7α-hydroxy-5β-cholan-24-oic acid (2). Dry methanol (65 μL, 1.6 mmol) and $LiBH_4$ (800 μL, 2 M in THF, 1.6 mmol) were added to a solution of **1** (100 mg, 0.23 mmol) in dry THF (2.5 mL) at 0 °C under argon and the resulting mixture was stirred for 5 h at 0 °C. The mixture was quenched by addition of NaOH (1 M, 460 μL) and then allowed to warm to room temperature. Ethyl acetate was added and the separated aqueous phase was extracted with ethyl acetate (3 ×10

mL). The combined organic phases were washed with water, dried (Na_2SO_4) and concentrated. HPLC purification on a Nucleodur 100-5 C18 (5μm; 10 mm i.d. x 250 mm) with $MeOH/H_2O$ (90:10) as eluent (flow rate 3 mL/min), gave 100 mg of 3β-azido-6α-ethyl-7α-hydroxy-5β-cholan-24-oic acid (**2**) (98%, t_R= 17.5 min). Selected ^1H NMR (400 MHz, CD_3OD): δ_H 3.89 (1H, br s, H-3), 3.65 (1H, br s, H-7), 2.30 (1H, m, H-23a), 2.20 (1H, m, H-23b), 0.96 (3H, d, *J* = 6.7 Hz, H_3-21), 0.95 (3H, s, H_3-19), 0.90 (3H, t, *J* = 7.3 Hz, H_3-26), 0.70 (3H, s, H_3-18); ^{13}C NMR (125 MHz, CD_3OD): δ_C 178.2, 71.4, 59.8, 57.4, 51.7, 43.7, 42.7, 42.0, 41.5, 41.0, 37.0, 36.8, 34.1, 32.4, 32.1, 31.9, 29.3, 28.1, 25.6, 24.5, 24.2, 23.3, 22.2, 18.8, 12.2, 12.0. HR ESIMS *m/z*444.3229 [M - H], $C_{26}H_{42}O_3N_3$requires 444.3226.

3β-azido-6α-ethyl-7α-hydroxy-5β-cholan-24-oyl taurine sodium salt (2a). Compound **2** (20 mg, 44.9 x 10^{-3}mmol) in DMF dry (3 mL) was treated with DMT-MM (37 mg, 0.13 mmol) and triethylamine (155 μL, 1.12 mmol) and the mixture was stirred at room temperature for 10 min. Then taurine (34 mg, 0.27 mmol) was added. After 24 h, the reaction mixture was concentrated under *vacuo*, dissolved in water (5 mL) and poured over a C18 silica gel column. Fraction eluted with H_2O/MeOH 99:1 was further purified by HPLC on a Phenomenex Luna C18 (5 μm; 4.6 mm i.d. x 250 mm) with $MeOH/H_2O$ (85:15) as eluent (flow rate 1 mL/min), to give 13 mg of compound **2a** (53%; t_R=21 min). Selected ^1H NMR (400 MHz CD_3OD): δ 3.89 (1H, br s, H-3), 3.65 (1H, br s, H-7), 3.59 (2H, t, *J*= 7.1 Hz, $-CH_2NH-$), 2.96 (2H, t, *J* = 7.1 Hz, $-CH_2SO_3H$), 0.97 (3H, d *J* = 6.5 Hz, H_3-21), 0.94 (3H, s, H_3-19), 0.91 (3H, t, *J* = 7.4 Hz, H_3-26), 0.69 (3H, s, CH_3-18); ^{13}C NMR (100 MHz, CD_3OD): δ_C 176.6, 71.4, 59.8, 57.3, 51.7, 51.5, 43.8, 42.7, 42.0, 41.5, 41.0, 37.0, 36.9, 36.5, 34.2, 34.1, 33.2, 32.1, 29.3, 28.1, 25.6, 24.5, 24.2, 23.3, 22.1, 18.9, 12.2, 12.0. HRESIMS *m/z* 551.3271 [M-Na], $C_{28}H_{47}N_4O_5S$ requires 551.3267.

3β-amino-6α-ethyl-7α-hydroxy-5β-cholan-24-oic acid (10). Compound **10** was prepared from **2** (20 mg, 45 x 10^{-3}mmol), by an analogous procedure to that detailed above for **9**. HPLC purification on a Nucleodur 100-5 C18 Isis (5 μm; 4.6 mm i.d. x 250 mm) with $MeOH/H_2O$ (70:30) as eluent

(flow rate 1 mL/min), gave 12 mg of 3β-amino-6α-ethyl-7α-hydroxy-5β-cholan-24-oic acid (**10**) as white solid (64%, t_R= 9.5 min). Selected ^{1}H NMR (400 MHz, CD_3OD): δ_H 3.67 (1H, br s, H-7), 3.48 (1H, br s, H-3), 2.49 (1H, dt, *J* = 15.6, 4.4 Hz, H-4a), 2.26 (1H, m, H-23a), 2.12 (1H, m, H-23b), 1.01 (3H, s, H_3-19), 0.96 (3H, d, *J* = 6.7 Hz, H_3-21), 0.92 (3H, t, *J* = 7.4 Hz, H_3-26), 0.70 (3H, s, H_3-18); ^{13}C NMR (100 MHz, CD_3OD): δ_C 178.4, 70.6, 57.4, 51.5, 49.0, 43.7, 42.3, 41.4, 41.1, 40.8, 37.4, 37.0, 34.5, 33.7, 33.3, 31.0, 29.1, 26.9, 24.4, 24.2, 23.5, 23.1, 22.0, 18.8, 12.2, 11.8. HR ESIMS *m/z* 418.3325 [M - H], $C_{26}H_{44}O_3N$ requires 418.3321.

3β-azido-6α-ethyl-7α-hydroxy-5β-cholan-24-ol (3). Compound **18** (50 mg, 0.11 mmol) was treated with MeONa (6 mg, 0.11 mmol) in methanol overnight to obtain inversion at C-6 giving methyl 3β-azido-6α-ethyl-7-keto-5β-cholan-24-oate (quantitative yield), that was subjected to next step without any purification. Dry methanol (30 μL, 0.77 mmol) and $LiBH_4$ (385 μL, 2 M in THF, 0.77 mmol) were added to a solution of the intermediate (50 mg, 0.11 mmol) in dry THF (5 mL) at 0 °C under argon and the resulting mixture was stirred for 5 h at 0 °C. The mixture was quenched by addition of NaOH (1 M, 220 μL) and then allowed to warm to room temperature. Ethyl acetate was added and the separated aqueous phase was extracted with ethyl acetate (3 ×10 mL). The combined organic phases were washed with water, dried (Na_2SO_4) and concentrated. HPLC purification on a Nucleodur 100-5 C18 (5μm; 10 mm i.d. x 250 mm) with MeOH/H_2O (90:10) as eluent (flow rate 3 mL/min), gave 45 mg of 3β-azido-6α-ethyl-7α-hydroxy-5β-cholan-24-ol (**3**) (95%, t_R= 45 min). Selected ^{1}H NMR (700 MHz, CD_3OD): δ_H 3.88 (1H, br s, H-3), 3.64 (1H, br s, H-7), 3.50 (2H, m, H_2-24), 2.24 (1H, dt, *J* = 15.1, 3.1 Hz, H-4a), 0.97 (3H, d, *J* = 6.4 Hz, H_3-21), 0.95 (3H, s, H_3-19), 0.91 (3H, t, *J* = 7.2 Hz, H_3-26), 0.70 (3H, s, H_3-18); ^{13}C NMR (175 MHz, CD_3OD): δ_C 71.5, 63.5, 59.6, 57.4, 51.4, 43.7, 42.5, 41.8, 41.3, 40.8, 36.8, 36.4, 34.0, 32.3, 31.9, 29.9, 27.9, 25.3, 24.0, 22.9, 21.9, 18.9, 12.0, 11.7. HR ESIMS *m/z* 432.3591 $[M + H]^+$, $C_{26}H_{46}O_2N_3$ requires 432.3590.

3β-amino-6α-ethyl-7α-hydroxy-5β-cholan-24-ol (11). Compound **11** was synthesized from **3** (20 mg, 46.4 x 10^{-3}mmol), by an analogous procedure to that detailed above for **9**. HPLC purification

on a Nucleodur 100-5 C18 Isis (5 μm; 4.6 mm i.d. x 250 mm) with MeOH/H_2O (70:30) as eluent (flow rate 1 mL/min), gave13 mg of 3β-amino-6α-ethyl-7α-hydroxy-5β-cholan-24-ol (**11**) as white solid (69%, t_R= 10 min). Selected ^{1}H NMR (700 MHz, CD_3OD): δ_H 3.68 (1H, br s, H-7), 3.51 (2H, m, H_2-24), 3.48 (1H, m, H-3), 2.49 (1H, dt, *J* = 15.1, 3.1 Hz, H-4a), 1.02 (3H, s, H_3-19), 0.97 (3H, d, *J* = 6.5 Hz, H_3-21), 0.94 (3H, t, *J* = 7.3 Hz, H_3-26), 0.71 (3H, s, H_3-18); ^{13}C NMR (175 MHz, CD_3OD): δ_C 70.4, 63.5, 57.2, 51.6, 49.0, 43.7, 42.1, 41.1, 41.2, 40.7, 36.9 (2C), 33.9, 33.0, 30.6, 30.0, 29.2, 26.9, 24.0, 23,7, 23.5, 23.4, 21.7, 18.8, 12.0, 11.9. HR ESIMS *m/z* 406.3689 $[M + H]^+$, $C_{26}H_{48}O_2N$ requires 406.3685.

3β-azido-6β-ethyl-7β-hydroxy-5β-cholan-24-ol (4). Compound **4** was synthesized from **18** (80 mg, 0.18 mmol), by an analogous procedure to that detailed above for **2**. HPLC purification on a Nucleodur 100-5 C18 (5μm; 10 mm i.d. x 250 mm) with MeOH/H_2O (90:20) as eluent (flow rate 3 mL/min), gave 35 mg of 3β-azido-6β-ethyl-7β-hydroxy-5β-cholan-24-ol (**4**) (45%, t_R= 38.8 min). Selected ^{1}H NMR (700 MHz, CD_3OD): δ_H 3.61 (1H, dd, *J* = 7.1, 5.0 Hz, H-7), 3.51 (2H, m, H_2-24), 3.44 (1H, m, H-3), 0.99 (3H, s, H_3-19), 0.97 (3H, t, *J* = 7.3 Hz, H_3-26), 0.96 (3H, d, *J* = 6.5 Hz, H_3-21), 0.71 (3H, s, H_3-18); ^{13}C NMR (175 MHz, CD_3OD): δ_C 75.0, 63.6, 59.0, 58.1, 57.1, 45.1, 44.4, 41.7 (2C), 41.2, 40.2, 36.9, 36.0, 34.4, 33.2, 31.2, 30.5, 29.6, 27.4, 27.0, 22.9, 22.8, 21.9, 19.3, 13.0, 12.3. HR ESIMS *m/z* 432.3593 $[M + H]^+$, $C_{26}H_{46}O_2N_3$ requires 432.3590.

3β-amino-6β-ethyl-7β-hydroxy-5β-cholan-24-ol (12). Compound **12** was synthesized from **4** (20 mg, 46.4 x 10^{-3}mmol), by an analogous procedure to that detailed above for **9**. HPLC purification on a Nucleodur 100-5 C18 Isis (5 μm; 4.6 mm i.d. x 250 mm) with MeOH/H_2O (70:30) as eluent (flow rate 1 mL/min), gave 3β-amino-6β-ethyl-7β-hydroxy-5β-cholan-24-ol (**12**) as white solid (13 mg, 69%, t_R= 8.5 min). Selected ^{1}H NMR (400 MHz, CD_3OD): δ_H 3.61 (1H, m, H-7), 3.50 (2H, m, H_2-24), 2.98 (1H, m, H-3), 1.00 (3H, s, H_3-19), 0.99 (3H, ovl, H_3-26), 0.96 (3H, d, *J* = 6.5 Hz, H_3-21), 0.70 (3H, s, H_3-18); ^{13}C NMR (175 MHz, CD_3OD): δ_C 74.5, 63.5, 57.7, 57.1, 48.8, 46.6, 43.9, 42.0,

40.9, 40.1, 39.0, 36.8, 35.9, 34.6, 33.0, 30.3, 29.9, 29.0, 27.4, 26.1, 23.1, 21.1, 20.7, 19.0, 12.1, 12.0. HR ESIMS *m/z* 432.3593 $[M + H]^+$, $C_{26}H_{46}O_2N_3$ requires 432.3590.

Methyl 3β-hydroxy-6β-ethyl-7-keto-5β-cholan-24-oate (19). To a solution of **17** (300 mg, 0.69 mmol) in dry pyridine (5 ml), tosyl chloride (663 mg, 3.5mmol) was added, and the mixture was stirred at room temperature for 6 h. It was poured into cold water (10 mL) and extracted with CH_2Cl_2 (3 × 10 mL). The combined organic layers were washed with saturated $NaHCO_3$ solution (10 mL), and water (10 mL), and then dried over anhydrous $MgSO_4$ and evaporated *in vacuo* to give 405 mg of methyl 3α-tosyloxy-6β-ethyl-7-keto-5β-cholan-24-oate (quantitative yield) in the form of colourless needles, that was subjected to next step without any purification. The intermediate and CH_3COOK (70 mg, 0.69 mmol) were dissolved in water (2 mL) and N,N'-dimethylformamide (DMF, 10 mL). After refluxing for 4 h, the solution was cooled at room temperature and then ethyl acetate and water were added. The separated aqueous phase was extracted with ethyl acetate (3 ×10 mL). The combined organic phases were washed with water, dried (Na_2SO_4) and evaporated to dryness. Purification by silica gel (hexane-ethyl acetate 8:2 and 0.5% TEA) gave 220 mg of **19** (74% over two steps). Selected ^1H NMR (400 MHz, $CDCl_3$): δ_H 3.86 (1H, br s, H-3), 3.55 (3H, s, $-COOCH_3$), 2.43 1H, t, J = 11.3 HZ, H-8), 2.24 (1H, m, H-23a), 2.12 (1H, m, H-23b), 1.12 (3H, s, H_3-19), 0.81 (3H, d, J = 6.4 Hz, H_3-21), 0.70 (3H, t, J = 7.2 Hz, H_3-26), 0.56 (3H, s, H_3-18); ^{13}C NMR (100 MHz, $CDCl_3$) δ_C216.2,174.4, 65.6, 60.7, 54.8, 51.2, 48.6, 45.5, 44.3, 42.7, 42.2, 38.6, 36.3, 35.7, 35.0, 30.8 (2C), 30.2, 27.9, 27.2, 26.1, 25.9, 24.7, 21.5, 18.1, 12.7, 11.8. HR ESIMS *m/z* 433.3321 $[M + H]^+$, $C_{27}H_{45}O_4$ requires 433.3318.

Methyl 3α-azido-6β-ethyl-7-keto-5β-cholan-24-oate (20). Compound **20** (185 mg, 81% over two steps) was synthesized from **19** (220 mg, 0.50 mmol), following the same synthetic procedures detailed above for **18**. Selected ^1H NMR (400 MHz, CD_3OD): δ_H 3.65 (3H, s, $-COOCH_3$), 3.31 (1H, ovl, H-3), 2.68 (1H, t, J = 11.3 Hz, H-8), 2.37 (1H, m, H-23a), 2.25 (1H, m, H-23b), 1.26 (3H, s, H_3-19), 0.95 (3H, d, J = 6.5 Hz, H_3-21), 0.83 (3H, t, J = 7.1 Hz, H_3-26), 0.71 (3H, s, H_3-18); ^{13}C

NMR (100 MHz, $CDCl_3$) δ_C 214.7,174.3, 61.7, 59.7, 54.5, 51.2, 49.4, 48.3, 45.2, 42.6, 42.2, 38.4, 35.4, 35.3, 35.2, 34.9, 30.7 (2C), 27.9, 26.4, 25.8, 25.6, 24.6, 21.1, 18.1, 12.8, 11.8. HR ESIMS *m/z* 458.3386 $[M + H]^+$, $C_{27}H_{44}O_3N_3$ requires 458.3383.

3α-azido-6α-ethyl-7-keto-5β-cholan-24-oic acid (5). Compound **5** was synthesized from **20** (100 mg, 0.22 mmol), by an analogous procedure to that detailed above for **1**. An analytical sample was obtained by HPLC on a Nucleodur 100-5 C18 Isis (5 μm; 4.6 mm i.d. x 250 mm) with $MeOH/H_2O$ (90:10) as eluent (flow rate 1 mL/min, t_R= 13.2 min). Selected ^1H NMR (400 MHz, CD_3OD): δ_H 3.31 (1H, m, H-3), 2.86 (1H, m, H-6), 2.52 (1H, t, *J* = 11.3 Hz, H-8), 2.34 (1H, m, H-23a), 2.21 (1H, m, H-23b), 1.28 (3H, s, H_3-19), 0.97 (3H, d, *J* = 6.5 Hz, H_3-21), 0.81 (3H, t, *J* = 7.3 Hz, H_3-26), 0.71 (3H, s, H_3-18); ^{13}C NMR (100 MHz, CD_3OD): δ_C 215.6, 178.2, 61.6, 56.2, 53.2, 52.2, 51.2, 50.3, 45.3, 40.3, 36.8, 36.5 (2C), 35.2, 32.3, 31.9, 29.3 (2C), 27.1, 25.6, 23.9, 22.9, 20.0, 18.8, 12.5, 12.3. HR ESIMS *m/z* 442.3071 [M - H], $C_{26}H_{40}O_3N_3$ requires 442.3070.

3α-amino-6α-ethyl-7-keto-5β-cholan-24-oic acid (13). Compound **13** was synthesized from **5** (35 mg, 79 x 10^{-3}mmol), by an analogous procedure to that detailed above for **9**. HPLC purification on a Nucleodur 100-5 C18 Isis (5 μm; 4.6 mm i.d. x 250 mm) with $MeOH/H_2O$ (60:40) as eluent (flow rate 1 mL/min), gave18 mg of 3β-ammino-6α-ethyl-7-keto-5β-cholan-24-oic acid (**13**) as white solid (54%, t_R= 21.3 min). Selected ^1H NMR (500 MHz, CD_3OD): δ_H3.04 (1H, m, H-3), 2.91 (1H, m, H-6), 2.55 (1H, t, *J* = 11.0 Hz, H-8), 2.26 (1H, m, H-23a), 2.11 (1H, m, H-23b), 1.31 (3H, s, H_3-19), 0.96 (3H, d, *J* = 6.5 Hz, H_3-21), 0.83 (3H, t, *J* = 7.5 Hz, H_3-26), 0.72 (3H, s, H_3-18); ^{13}C NMR (100 MHz, CD_3OD): δ_C 214.8, 178.1, 56.6, 52.5, 51.4, 51.2, 50.6, 50.1, 45.1, 43.3, 39.9, 36.1 (2C), 34.1, 31.9, 31.6, 31.4, 29.0, 27.8, 25.6, 23.1, 22.4, 19.3, 18.5, 12.0, 11.9. HR ESIMS *m/z* 416.3171 [M - H], $C_{26}H_{42}O_3N$ requires 416.3165.

3α-azido-6α-ethyl-7α-hydroxy-5β-cholan-24-oic acid (6). Compound **6** was synthesized from **5** (50 mg, 0.11 mmol), by an analogous procedure to that detailed above for **2**. HPLC purification on a Nucleodur 100-5 C18 Isis (5 μm; 4.6 mm i.d. x 250 mm) with $MeOH/H_2O$ (90:10) as eluent (flow

rate 1 mL/min), gave 44 mg of 3α-azido-6α-ethyl-7α-hydroxy-5β-cholan-24-oic acid (**6**) (90%, t_R= 18 min). Selected ^{1}H NMR (400 MHz, CD_3OD): δ_H 3.66 (1H, br s, H-7), 3.11 (1H, m, H-3), 2.34 (1H, m, H-23a), 2.19 (1H, m, H-23b), 0.96 (3H, d, *J* = 6.3 Hz, H_3-21), 0.93 (3H, s, H_3-19), 0.91 (3H, t, *J* = 7.2 Hz, H_3-26), 0.69 (3H, s, H_3-18); ^{13}C NMR (100 MHz, CD_3OD): δ_C 178.3, 71.0, 63.1, 57.4, 51.6, 47.2, 43.7, 43.1, 41.5, 40.9, 36.9 (2C), 36.7, 34.5, 32.4, 32.0, 30.9, 29.3, 27.8, 24.6, 23.8, 23.5, 21.9, 18.8, 12.3, 12.0. HR ESIMS *m/z* 444.3229 [M - H], $C_{26}H_{42}O_3N_3$ requires 444.3226.

3α-amino-6α-ethyl-7α-hydroxy-5β-cholan-24-oic acid (14). Compound **14** was synthesized from **6** (25 mg, 56,6 x 10^{-3}mmol), by an analogous procedure to that detailed above for **9**. HPLC purification on a Nucleodur 100-5 C18 Isis (5 μm; 4.6 mm i.d. x 250 mm) with MeOH/H_2O (73:27) as eluent (flow rate 1 mL/min), gave 12 mg of 3α-ammino-6α-ethyl-7α-hydroxy-5β-cholan-24-oic acid (**14**) as white solid (50%, t_R= 13 min). Selected ^{1}H NMR (500 MHz, CD_3OD): δ_H 3.65 (1H, br s, H-7), 2.87 (1H, m, H-3), 2.28 (1H, m, H-23a), 2.14 (1H, m, H-23b), 0.97 (3H, s, H_3-19), 0.95 (3H, d, *J* = 6.2 Hz, H_3-21), 0.92 (3H, t, *J* = 7.5 Hz, H_3-26), 0.71 (3H, s, H_3-18); ^{13}C NMR (100 MHz, CD_3OD): δ_C 178.2, 71.2, 57.6, 53.0, 51.7, 47.3, 43.7, 43.1, 41.6, 41.1, 37.3, 37.0, 36.1, 34.6, 33.9, 31.6, 31.4, 30.7, 29.4, 23.8, 23.5, 24.6, 22.0, 18.5, 12.2, 12.0. HR ESIMS *m/z* 418.3323 [M - H], $C_{26}H_{44}O_3N$ requires 418.3321.

3α-azido-6α-ethyl-7α-hydroxy-5β-cholan-24-ol (7). Compound **7** was synthesized from **20** (45 mg, 98.5 x 10^{-3}mmol), by analogous procedures to that detailed above for **3**. HPLC purification on a Nucleodur 100-5 C18 (5 μm; 10 mm i.d. x 250 mm) with MeOH/H_2O (85:15) as eluent (flow rate 3 mL/min), gave 40 mg of 3α-azido-6α-ethyl-7α-hydroxy-5β-cholan-24-ol (**7**) (94%, t_R= 32.5 min). Selected ^{1}H NMR (400 MHz, CD_3OD): δ_H3.65 (1H, br s, H-7), 3.50 (2H, m, H_2-24), 3.11 (1H, m, H-3), 0.96(3H, d, *J* = 6.5 Hz, H_3-21), 0.93 (3H, s, H_3-19), 0.91 (3H, t, *J* = 7.2 Hz, H_3-26), 0.70 (3H, s, H_3-18); ^{13}C NMR (100 MHz, CD_3OD): δ_C71.1, 63.7, 63.2, 57.7, 51.1, 47.4, 43.9, 43.3, 41.6, 41.2, 37.2, 37.0 (2C), 34.3, 33.5, 31.2, 30.5, 29.5, 28.0, 24.6, 23.7, 23.6, 22.0, 19.3, 12.4, 12.1. HR ESIMS *m/z* 432.3593 $[M + H]^+$, $C_{26}H_{46}O_2N_3$ requires 432.3590.

3α-amino-6α-ethyl-7α-hydroxy-5β-cholan-24-ol (15). Compound **15** was synthesized from **7** (20 mg, 46.4 x 10^{-3}mmol), by an analogous procedure to that detailed above for **9**. HPLC purification on a Nucleodur 100-5 C18 Isis (5 μm; 4.6 mm i.d. x 250 mm) with MeOH/H_2O (70:30) as eluent (flow rate 1 mL/min), gave 10 mg 3α-amino-6α-ethyl-7α-hydroxy-5β-cholan-24-ol (**15**) as white solid (53%, t_R= 13.7 min). Selected ^{1}H NMR (400 MHz, CD_3OD): δ_H 3.68 (1H, br s, H-7), 3.50 (2H, m, H_2-24), 2.87 (1H, m, H-3), 0.97 (3H, s, H_3-19), 0.96 (3H, ovl, H_3-21), 0.92 (3H, t, J = 7.3 Hz, H_3-26), 0.72 (3H, s, H_3-18); ^{13}C NMR (100 MHz, CD_3OD): δ_C 71.2, 63.6, 57.6, 53.0, 51.7, 47.4, 43.7, 43.1, 41.6, 41.1, 37.3, 37.0, 36.6, 34.6, 33.9, 33.2, 30.7, 30.3, 29.4, 24.6, 23.8, 23.5, 22.0, 19.2, 12.2, 12.0. HR ESIMS *m/z* 406.3687 $[M + H]^+$, $C_{26}H_{48}O_2N$ requires 406.3685.

3α-azido-6β-ethyl-7β-hydroxy-5β-cholan-24-ol (8). Compound **8** was synthesized from **20** (50 mg, 0.11 mmol) by an analogous procedure to that detailed above for **2**. HPLC purification on a Nucleodur 100-5 C18 Isis (5 μm; 4.6 mm i.d. x 250 mm) with MeOH/H_2O (90:20) as eluent (flow rate 1 mL/min), gave 22 mg of 3α-azido-6β-ethyl-7β-hydroxy-5β-cholan-24-ol (**8**) (46%, t_R= 46.2 min). Selected ^{1}H NMR (500 MHz, CD_3OD): δ_H 3.70 (1H, dd, J = 10.2, 6.0 Hz, H-7), 3.50 (2H, m, H_2-24), 3.33 (1H, ovl, H-3), 1.01 (3H, s, H_3-19), 0.98 (3H, ovl, H_3-21), 0.97 (3H, ovl, H_3-26), 0.72 (3H, s, H_3-18); ^{13}C NMR (125 MHz, CD_3OD): δ_C 75.1, 63.6, 62.0, 57.4, 56.5, 51.3, 45.5, 44.8, 42.1, 41.4, 40.4, 36.9 (2C), 36.4, 35.9, 33.2, 30.3, 29.7, 28.4, 27.2, 26.0, 23.3, 22.1, 19.4, 14.7, 12.7. HR ESIMS *m/z* 432.3594 $[M + H]^+$, $C_{26}H_{46}O_2N_3$ requires 432.3590.

3α-amino-6β-ethyl-7β-hydroxy-5β-cholan-24-ol (16). Compound **16** was synthesized from **8** (15 mg, 34.8 x 10^{-3}mmol), by an analogous procedure to that detailed above for **9**. HPLC purification on a Nucleodur 100-5 C18 Isis (5 μm; 4.6 mm i.d. x 250 mm) with MeOH/H_2O (77:23) as eluent (flow rate 1 mL/min), gave 9.5 mg of 3α-amino-6β-ethyl-7β-hydroxy-5β-cholan-24-ol (**16**) as white solid (68%, t_R= 14.2 min). Selected ^{1}H NMR (400 MHz, CD_3OD): δ_H 3.73 (1H, dd, J = 10.3, 5.9 Hz, H-7), 3.50 (2H, m, H_2-24), 2.47 (1H, m, H-3), 1.03 (3H, s, H_3-19), 0.98 (3H, ovl, H_3-21), 0.98 (3H, ovl, H_3-26), 0.74 (3H, s, H_3-18); ^{13}C NMR (100 MHz, CD_3OD): δ_C 75.4, 63.6, 58.0, 57.1, 56.5, 51.7,

46.0, 45.2, 41.5, 41.3, 40.2, 37.4, 36.6, 36.0 (2C), 33.3, 30.1, 29.5, 29.4, 28.2, 26.0, 23.4, 21.9, 19.2, 14.7, 12.7. HR ESIMS *m/z* 405.3610 $[M + H]^+$, $C_{26}H_{48}O_2N$ requires 405.3606.

2.1 Supplementary Figure 1

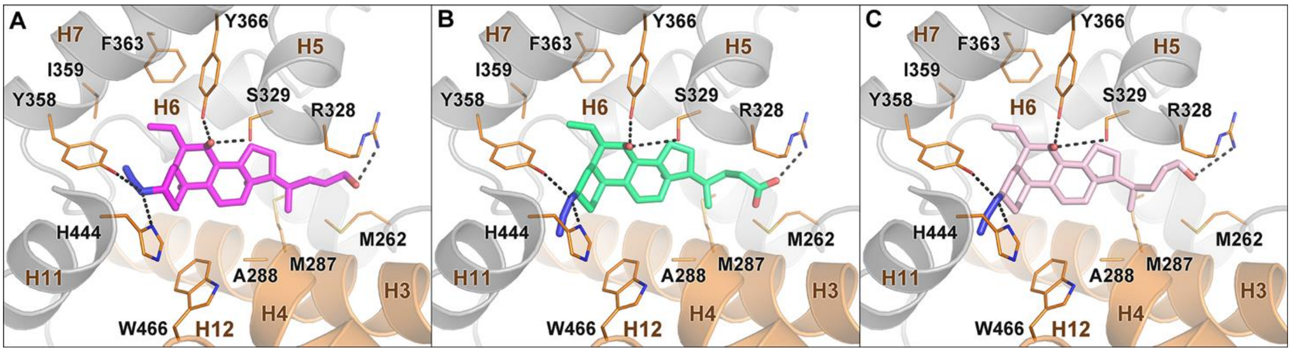

Supplementary Figure 1. (A) Binding mode of compound **3** (magenta sticks), (B) compound **6** (green sticks) and (C) compound **7** (pink sticks) in the FXR-LBD. FXR is shown as orange (helices H3, H4, and H12) and gray cartoons. Amino acids involved in ligand binding are shown as orange sticks. Hydrogens are omitted for clarity.
